# Expression pattern of CD11c on lung immune cells after disseminated murine cytomegalovirus infection

**DOI:** 10.1186/s12985-017-0801-x

**Published:** 2017-07-18

**Authors:** Yi Liao, Xinglou Liu, Yuan Huang, Heyu Huang, Yuanyuan Lu, Yanan Zhang, Sainan Shu, Feng Fang

**Affiliations:** Department of Pediatrics, Tongji Hospital, Tongji Medical College, Huazhong University of Science and Technology, Wuhan, Hubei 430030 China

**Keywords:** Murine cytomegalovirus, CD11c, CD8^+^T cells, CD11c^hi^MHC-II^hi^, NK cells, B220, B220^+^CD11c^int^ NK cells, NKp46

## Abstract

**Background:**

Cytomegalovirus (CMV) infection occurs frequently and is widespread globally. Numerous studies have shown that various types of immune cells play roles in mediating the response to CMV infection. CD11c, a commonly used dendritic cell (DC) marker, is expressed by other immune cells as well, such as T cells. This study analyzed the immune cells that express CD11c and monitored the expression level of their specific cell surface markers in the lung following a disseminated murine (M)CMV infection.

**Methods:**

Mouse models of disseminated MCMV infection were used; uninfected and lipopolysaccharide (LPS)-treated mice were used as controls. At 1, 3 and 7 days following infection, single cell suspensions prepared from freshly digested lung tissue were stained for CD11c, CD86 and MHC II. Stained cells were analyzed using flow cytometry. Peripheral blood and single cell suspensions from spleen were sorted as well. Then these cells were subjected to analyze the CD11c expression pattern on natural killer (NK) cells and T cells.

**Results:**

This assay showed that after MCMV infection, the expression of CD86 on pulmonary CD11c^hi^MHC-II^hi^ cells (encompassing conventional DCs) was higher at 3 days post-infection than at 1 or 7 days post-infection, accompanied by a downregulation of MHC II. In addition, expression of CD11c was greatly increased in the MCMV infection group at 7 days post infection. This study also detected a large population of cells displaying an intermediate level of expression of CD11c (CD11c^int^); these cells were in the MCMV groups exclusively, and were subsequently identified as CD8^+^ T cells. In lung, spleen and blood, different proportions of CD11c^int^ cells among the NK cell and T cell populations were observed between the BALB/c and C57BL/6 mice with or without MCMV infection. The expression level of NKp46 in NK cells dropped to a lower level after MCMV infection.

**Conclusions:**

The findings collectively indicate that CD11c^int^CD8^+^ T cells might play a key role in anti-MCMV adaptive immune response in lungs, as well as in spleen and blood. B220^+^CD11c^int^ NK cells might be a more effective type of NK cell, participating in anti-MCMV infection. The downregulation of NKp46, in particular, might be linked with the immune evasion of MCMV.

**Electronic supplementary material:**

The online version of this article (doi:10.1186/s12985-017-0801-x) contains supplementary material, which is available to authorized users.

## Background

Cytomegalovirus (CMV) is a beta-herpes virus with high infection rate in humans, and the infection can be lifelong, without acute disease in healthy hosts. Unfortunately, reactivation from latency is a major cause of morbidity and mortality in immunocompromised hosts. Murine (M)CMV recapitulates many of the physiopathological characteristics of human CMV infection, and as such can serve as a model for studying the immunobiology of CMV infection of humans. In general, the immune response against CMV involves various types of cells, such as natural killer (NK) cells and T cells, which participate in restricting the primary infection and dampening reactivation [1]. The activation of both CD8^+^ T cells and NK cells heavily relies on their cross-talk with dendritic cells (DCs) [2], which serve as a link between innate and adaptive immunity and also play a significant role in mediating the immune response to CMV infection. DCs can be divided into two major subsets: conventional DCs (cDCs) and plasmacytoid DCs (pDCs), which are both present in mouse lungs [3–5].

During infection, DCs recognize CMV double-stranded DNA mainly through the Toll-like receptor 9, which triggers a distinct signaling pathway and results in the production of inflammatory cytokines and type I interferon [6, 7]. In addition, upon activation, DCs up-regulate the production of co-stimulatory molecules and change the expression of surface chemokine receptors in order to migrate to the lymphoid tissues. In the spleen and lymph nodes, DCs up-regulate MHC molecules. After encountering antigen-presenting mature DCs, naïve T cells are activated and become antigen-specific T effector cells, a process that is crucial for the initiation of adaptive immunity. Mechanistic studies have also shown that CMV can evade the immune system by paralyzing DCs, specifically by impairing the function and cytokine secretion capacity of monocyte-derived DCs (moDCs), rendering them incapable of inducing proliferation of T-cells [8–11].

CD11c, also known as integrin α_x_, is a commonly used marker of DCs; however, it can also be expressed on T cells, NK cells, monocytes, macrophages, neutrophils and some B cells [12]. An increasing number of studies have aimed to characterize the relation of CD11c with functions of the various immune cells, recognizing this factor’s importance beyond a cell phenotype marker. Indeed, it has been demonstrated that mouse DCs strongly downregulate CD11c expression upon activation and that this process is triggered by Toll-like receptor signaling [13]. In addition, CD11c^+^CD8^+^ T cells have been reported as remarkably efficient producers of IFN-γ and to play an important role in mediating its related cytotoxic effects, ultimately aiding in viral clearance and tumor regression [14, 15]. In a comparative analysis of CD11c^+^ cells with CD11c^−^ liver NK cells, the former displayed an activated phenotype and enhanced effector functions, facilitating contributions to early hepatic IFN-γ production during adenovirus infection [16]. The collective findings reported in the literature have suggested that the expression pattern of CD11c on immune cells might be related with their activation and function.

In the study reported herein, in vivo experiments were used to address the expression pattern of CD11c on various immune cells in the lung following dissemination of MCMV infection, with the expression level of the specific cell surface markers monitored during the infection.

## Methods

### Animals and MCMV infection

Female 4-week-old BALB/c specific pathogen-free mice, weighing 8–10 g, were purchased from a local experimental animal research center (Wuhan, China). All mice were fed normal diet for 3 days and then randomly divided into the following three groups (*n* = 12 each): MCMV infection, lipopolysaccharide (LPS) stimulation and untreated control. The respective treatments included intraperitoneal inoculation with 200 μL salivary gland homogenate containing 5 × 10^3^ plaque forming units (PFUs) of MCMV Smith strain, with *Escherichia coli* LPS (0.25 μg/g; Sigma-Aldrich, USA) or DMEM (Gibco, USA). At 1, 3 and 7 days after injection, lungs were harvested aseptically under ether anesthesia.

### Preparation of pulmonary single-cell suspension

After carefully discarding the thoracic lymph nodes and thymus, the lungs were dissected and submerged in ice-cold tissue culture medium (RPMI-1640 supplemented with 5% fetal calf serum, 2-mercaptoethanol and penicillin/streptomycin; procured from Gibco, Hyclone and Sigma-Aldrich, USA, respectively). Following thorough mincing, the tissues were treated with 1 mg/mL collagenase type II (Gibco) and 0.02 mg/mL DNase I (Roche Diagnostics Corporation, Switzerland). The samples were then incubated in a humidified 5% CO_2_ incubator at 37 °C for 30–45 min, with mechanical shaking every 15 min to help digestion. Next, the samples were vigorously agitated using glass pipettes, treated with more freshly prepared 1 mg/mL collagenase type II and 0.02 mg/mL DNase I, and incubated for an additional 15 min. The digested tissues were then centrifuged, resuspended in PBS containing 10 mM EDTA, and incubated for 5 min on a shaker at room temperature. Following a 7-min lysis of red blood cells, the samples were washed in PBS and RPMI-1640, and passed through a 75 μm cell-strainer. The final samples were resuspended in RPMI-1640 with a drop of fetal calf serum, and incubated on ice until processing for immunofluorescent labeling.

### Immunolabeling of single-cell suspension for flow cytometry

100 μL of sample, containing of 1 × 10^6^ cells, was first incubated with Fc receptor- blocking antibody (anti-CD16/CD32; BD Pharmingen, USA) for 5 min to reduce non-specific binding. Next, the sample was labeled for 20 min in the dark at 4 °C, with the following anti-CD primary antibodies: PE hamster anti-mouse CD11c (BD Pharmingen, USA), FITC rat anti-mouse CD86 (BD Pharmingen), APC anti-mouse MHC Class II (eBiosciences, USA). Labeled cells were washed three times with PBS supplemented with 2% bovine serum albumin (Sigma-Aldrich) and 0.1% NaN_3_, and fixed. Flow cytometric analysis was performed on a Becton-Dickinson LSRII (USA).

### Validation of disseminated MCMV infection

Spleen and small lung-portion specimens obtained from each mouse were stored at −80 °C until analysis. MCMV infections were detected to verify the MCMV infection group by using qPCR to amplify the MCMV *gB* gene DNA (at 1 day post infection, dpi) and plaque assay to detect MCMV infection viral titers (at 3 and 7 dpi). For plaque assay, the organs were first homogenized in 1 mL of DMEM (supplemented with 4% fetal calf serum) and diluted in 1:10 steps. Diluted homogenates were then layered on murine embryonic fibroblasts (MEFs) and incubated at 37 °C for 60 min, after which the supernatants were discarded and cells were overlaid with 1% carboxymethylcellulose (Sigma-Aldrich)-DMEM containing 4% fetal calf serum to prevent secondary viral spread. Finally, the cells were incubated at 37 °C for 5–7 days, when viral titers were determined.

### Assessment of cell types among the increased CD11c^int^ cells

At 7 dpi, pulmonary single-cell suspension was obtained and labeled using the method described above but with the following labeling antibodies: APC anti-mouse CD11c, FITC anti-mouse MHC Class II, PE anti-mouse NKp46, PE/Cy7 anti-mouse CD19, PerCP/Cy5.5 anti-mouse CD3ε, PE anti-mouse F4/80, PE/Cy7 anti-mouse Ly-6G, PerCP/Cy5.5 anti-mouse Siglec H (all from BioLegend, USA), FITC anti-mouse CD4 and PE anti-mouse CD8a (both from eBioscience).

### Analysis of MCMV-specific CD8^+^ T cells

Tetramer complexes (produced by HelixGen Company, China) of APC-labeled mouse H-2D^d^ incorporating the AGPPRYSRI nonapeptide (encoded by the MCMV gene *m164*) were added to the 7-dpi pulmonary single-cell suspensions, along with FITC anti-mouse CD8a, PerCP/Cy5.5 anti-mouse CD3ε and PE anti-mouse CD11c (all from BioLegend or eBioscience).

### Analysis of CD11c^int^ CD8^+^ T cells in spleen and blood

At 7 dpi, peripheral blood was collected in 5 mM EDTA-containing tubes, to prevent clotting. After that, the spleen was obtained and passed through a 70 μM cell strainer (by mechanical means, utilizing the thumb-piece of a plunger removed from a 1 mL syringe). Following two rounds of red blood cell lysis, each spleen sample was then washed in PBS and resuspended in RPMI-1640 supplemented with a drop of fetal calf serum. Aliquots (100 μL each) of spleen single-cell suspension (1 × 10^6^ cells) and peripheral blood were labeled using the method described above but with the following labeling antibodies: APC anti-mouse CD11c, FITC anti-mouse CD8a, PE anti-mouse CD4 and PerCP/Cy5.5 anti-mouse CD3ε (all from BioLegend or eBioscience). After labeling, the spleen samples were washed and fixed, while the peripheral blood samples were subjected to two rounds of red blood cell lysis and then washed and fixed.

### Expression pattern of CD11c and B220 on NK cells

At 1, 3, 5 and 7 dpi, pulmonary and spleen single-cell suspensions were obtained and labeled using the method described above with the following labeling antibodies: PE anti-mouse CD11c, FITC anti-mouse B220, PerCP/Cy5.5 anti-mouse CD3ε and PE/Cy7 anti-mouse NKp46 (all antibodies from BioLegend).

### Post-acquisition data analysis

Analysis of flow cytometry data was performed on the WinMDI (version 2.09) software and FlowJo V10 software (TreeStar, USA). Values are presented as mean and standard deviation (SD). Statistical analyses were carried out using the Mann-Whitney and Kruskal-Wallis tests. *P* values <0.05 were considered statistically significant.

## Results

### Validation of disseminated MCMV infection

Disseminated MCMV infection was confirmed in all MCMV-infected mice. MCMV *gB* gene DNA (copies·μg^−1^) in lungs and spleens at 1 dpi were (4.07 ± 2.29) × 10^2^ and (9.15 ± 0.97) × 10, respectively. At 3 dpi, the MCMV infection viral titers (PFU·ml^−1^·mg^−1^) in lung and spleen tissues were 1.1 ± 0.7 and 3.3 ± 0.4, respectively, and at 7 dpi were 11.8 ± 2.2 and 1.3 ± 0.4, respectively.

### Appearance of CD11c^hi^MHC-II^hi^ cells in lungs

**Fig. 1:**
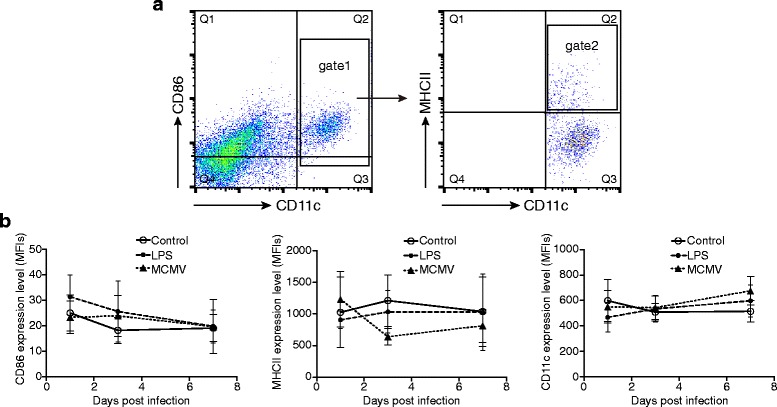
Analysis of cDCs (CD11c^hi^MHCII^hi^ cells) in lungs. **a** Identification of lung cDCs: Representative dot-plots of lung samples are shown. First, CD11c^hi^ cells were gated (gate 1), followed by the CD11c^hi^MHCII^hi^ cells (gate 2). **b** Analysis of surface markers of CD11c^hi^MHCII^hi^ cells: The expression level of cDC surface markers CD86, MHC II and CD11c in control, LPS and MCMV groups at 1, 3 and 7 dpi are shown (*n* = 4 mice/group at each time point, results are representative of four independent experiments). *Graphs* represent mean ± SD

We investigated changes in the expression of surface molecules on lung CD11c^hi^MHC-II^hi^ cells, which encompass cDCs [12] (Fig. 1a), specifically focusing on the levels of CD11c, CD86 and MHC II (Fig. 1b). The MHC class II molecules are constitutively expressed on antigen-presenting cells such as DCs, and their main function is to present processed antigens to CD4^+^ T lymphocytes. On the other hand, CD86 is a co-stimulatory molecule involved in the activation of naïve T cells. This study shows that, in comparison with the control group, in MCMV-infected mice, the expression level of CD86 was the highest and that of MHC II was the lowest at 3 dpi. In LPS-treated mice, the expression of CD86 reached maximum at 1 dpi, to levels higher than the control group. Subsequently, CD86 expression levels were restored gradually to levels similar to the control group. In contrast, MHC II expression in the LPS-treated mice was lower than that in the control group at 1 dpi and 3 dpi. The MHC II levels were restored to control levels by 7 dpi. Finally, CD11c, a type I transmembrane integrin (β2) used commonly as a DC marker, was found to be unaltered at both 1 dpi and 3 dpi in the MCMV mice, in comparison with the control group, but the expression was strongly increased at 7 dpi. In the LPS-treated group, the expression of CD11c was found to be below the control level at 1 dpi, but was restored subsequently.

### Production of CD11c^int^ cells in MCMV-infected lungs

**Fig. 2:**
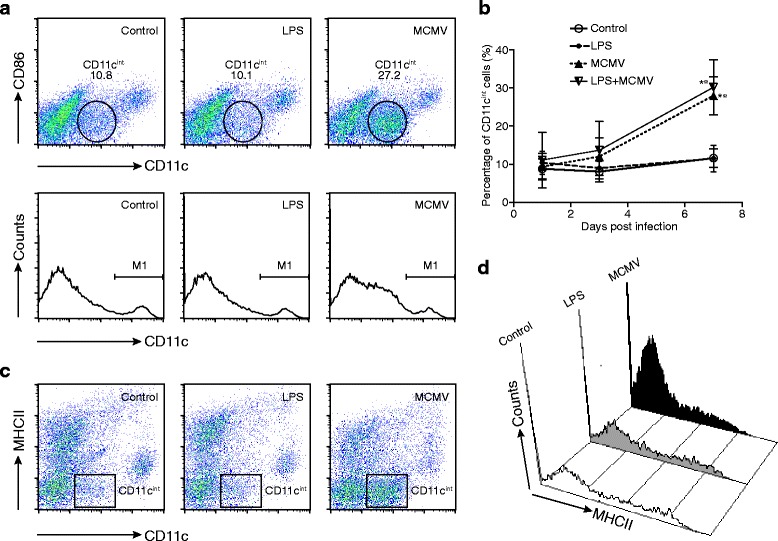
Production of CD11c^int^ cells in MCMV-infected lungs. **a** Dot-plots of lung samples isolated from control, LPS and MCMV groups at 7 days post infection (dpi) are shown. Relative expression of CD11c (X axis) and CD86 (Y axis) is depicted. *Cells inside the circles* represent the CD11c^int^ population, with low expression of CD86. Histogram of CD11c within each group is shown in the substratum. Both control and LPS groups show two cell populations (CD11c^lo^ and CD11c^hi^), while MCMV group shows an extra population of CD11c^int^ cells. **b** The percentage of CD11c^int^ cells in control, LPS, MCMV and LPS + MCMV groups at 1, 3 and 7 dpi is shown (*n* = 4 mice/group at each time point, results are representative of four independent experiments). *Graphs* represent mean ± SD. * *P* < 0.05 compared to controls; ☆ *P* < 0.05 compared to LPS. **c**. Dot-plots of lung samples isolated from control, LPS and MCMV groups at 7 dpi are shown, comparing the expression of CD11c (X axis) and MHC II (Y axis). The *cells in the rectangles* represent the expanded CD11c^int^ population. **d** Histograms of cell surface MHC II expression in the CD11c^int^ population in each group at 7 dpi are shown. The expansion of the CD11c^int^ population in MCMV group is mainly due to an increase in the proportion of MHC II^lo^ cells

During analysis of the data obtained from the flow cytometry assay, a large population of cells with intermediate expression of CD11c (CD11c^int^) was identified, the proportion of which increased markedly at 7 dpi in MCMV-infected mice, with no significant expansion in either the control or the LPS group (Fig. 2a). Although the assays reported here did identify a slight increase in the size of the CD11c^int^ population in MCMV-infected mice at 3 dpi, in comparison to the control group, this difference was much larger and significant at 7 dpi. To confirm the appearance of CD11c^int^ cells as part of the physiological response to MCMV infection alone, MCMV mice were treated with LPS 3 h after infection, and assayed by flow cytometry for CD11c^int^ cells. Interestingly, the same results as described above were obtained (Fig. 2b).

Subsequently, we further explored the properties of the CD11c^int^ cells and found that they express no to very-low levels of CD86 and MHC II (CD11c^int^MHC II^−/lo^CD86^−/lo^) (Fig. 2c and d).

### Types of cells represented among the increased CD11c^int^ cells

**Fig. 3:**
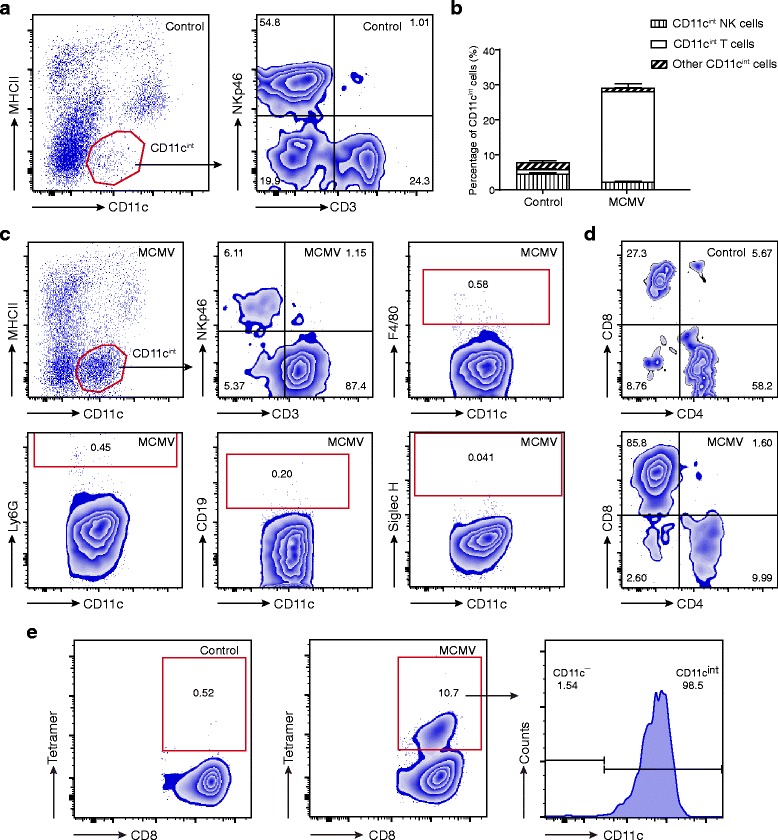
Types of cells represented among the increased CD11c^int^ cells. **a** Lung samples from the control group are shown. CD11c^int^ cells were gated and presented based on their expression of CD3 and NKp46. **b** The percentage of CD11c^int^ cells in control and MCMV groups at 7 days post infection (dpi) is shown in the form of constituent ratio (*n* = 4 mice/group, results are representative of two independent experiments). Data are expressed as mean ± SD. **c** Lung samples from MCMV group at 7 dpi are shown. CD11c^int^ cells were gated and presented. *Cells in the red rectangles* represent the cells with positivity for F4/80, Ly6G, CD19 and Siglec H, respectively. Nearly 90% of CD11c^int^ cells were CD3^+^NKp46^−^ T cells. **d** CD11c^int^ T cells in the control and MCMV lungs at 7 dpi are shown based on their expression of CD4 and CD8. **e** CD8^+^T cells in the control and MCMV lungs at 7 dpi are shown. Tetramer^+^ CTLs are gated using *red rectangles* and nearly all tetramer^+^ CTLs are CD11c^int^ cells

In order to ascertain which cell types represent the increased CD11c^int^ cells in MCMV-infected lungs at 7 dpi, labeling antibodies of NKp46, CD3ε, CD19, Ly6G, F4/80 and Siglec H were used to identify the NK cells, T cells, B cells, granulocytes, macrophages and pDCs, respectively. The result shows that NK cells (NKp46^+^CD3ε^−^) and T cells (CD3ε^+^NKp46^−^) were the two major populations of these CD11c^int^ cells (Fig. 3a) in the control group. After MCMV infection, however, the lung T cells increased markedly and accounted for the vast majority of CD11c^int^ cells at 7 dpi (Fig. 3b and c). In contrast, the percentage of CD11c^int^ NK cells was significantly decreased at 7 dpi. To further distinguish these CD11c^int^ T cells, CD4 and CD8a labeling antibodies were added, and it was found that CD8a^+^ cytotoxic T lymphocytes (CTLs) accounted for the increased CD11c^int^ cells (Fig. 3d).

### Analysis of MCMV-specific CD8^+^ T cells in MCMV-infected lungs

Considering the large number of CD11c^int^CD8^+^ T cells detected in MCMV-infected lungs at 7 dpi, we further explored whether these increased cells were MCMV-specific cells. Analysis with APC-labeled MHC tetramers indicated that approximately 10% of CD8^+^ T cells in MCMV-infected lungs were m164 peptides-specific cells (compared to <1% in the control group). Nearly all the tetramer^+^CD8^+^ T cells were found to be CD11c^int^ cells (Fig. 3e).

### Distribution of CD11c^int^ cells in T cells

**Fig. 4:**
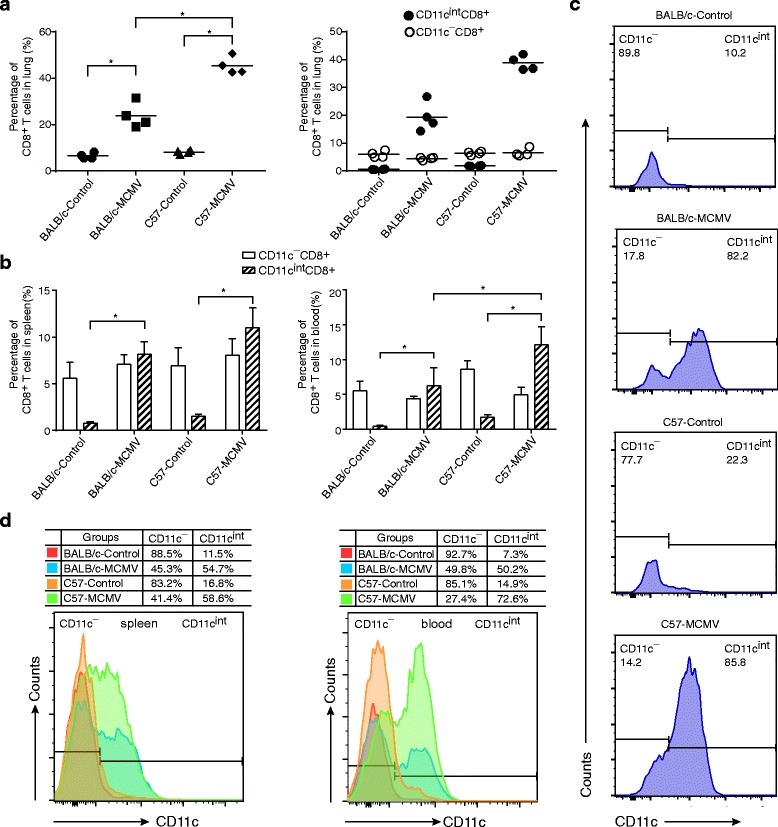
Distinct distribution of CD11c expression on CTLs of BALB/c and C57BL/6 mice. Both BALB/c and C57BL/6 mice were inoculated intraperitoneally with 5 × 10^3^ PFU of MCMV Smith strain. DMEM was injected into the (uninfected) control groups following the same study design. Lung, spleen and peripheral blood samples were harvested from each mouse and analyzed at 7 days post infection (dpi). **a** The percentages of CD8^+^ T cells in lungs of control and MCMV groups are shown. Total CTLs of each group are shown first, and then CD11c^−^ and CD11c^int^CTLs are shown separately (*n* = 4 mice/group, results are representative of two independent experiments). * *P* < 0.05. **b**. The percentages of CD11c^−^ and CD11c^int^ CTLs in spleen and peripheral blood of control and MCMV groups are shown (*n* = 4 mice/group, results are representative of two independent experiments). * *P* < 0.05. **c** Lung CD8^+^ T cells of each group are shown by histogram, based on their CD11c expression. **d** Spleen and blood CD8^+^ T cells of each group are shown by histogram, based on their CD11c expression. The proportions of CD11c^−^ and CD11c^int^ T cells in each group are shown in the table above the histogram

In both uninfected control and MCMV groups, the number of CD4^+^ T cells in lung remained stable with no or low CD11c surface expression (Additional file 1). In contrast, CD8^+^ T cells consist of CD11c^−^ and CD11c^int^ subpopulations. The data from both BALB/c mice and C57BL/6 mice showed that CD11c^−^ cells constituted the majority of CTLs in the control lungs, and a slightly higher proportion was observed in the BALB/c mice (90% vs C57BL/6 mice: 80%). After MCMV infection, significant expansion of CD11c^int^ CTLs was observed in the BALB/c as well as the C57BL/6 mice, and was found to constitute the increased CD11c^int^ cells of lung, indicating that CD11c^int^ cells emerged as the major portion of CTLs (approx. 80% ~ 85%; Fig. 4a and c). MCMV induced an obviously higher CTL level in the C57BL/6 mice than the BALB/c mice, and CD11c^int^ CTLs in particular were responsible for this difference (Fig. 4a). No change was observed in the percentage of CD11c^−^ CTLs in mice infected with MCMV compared to uninfected control.

To determine whether the expression change of CD11c on CTLs was typical of lung, we next examined spleen and peripheral blood and similar results were observed (Fig. 4b and d). Both spleen and peripheral blood showed significant expansions of CD11c^int^ CTLs after MCMV infection at 7 dpi, and higher levels can be found in the C57BL/6 mice (Fig. 4b). The difference between the three tissues (lung, spleen and blood) existed in the proportions of CD11c^int^ cells in the CTLs (Fig. 4c and d). In lung, the CD11c^int^ cells accounted for 80% ~ 85% of the CTLs after MCMV infection, but in spleen and blood the proportion of CD11c^int^ CTLs was only 50% ~ 70%.

### Expression pattern of CD11c in NK cells

**Fig. 5:**
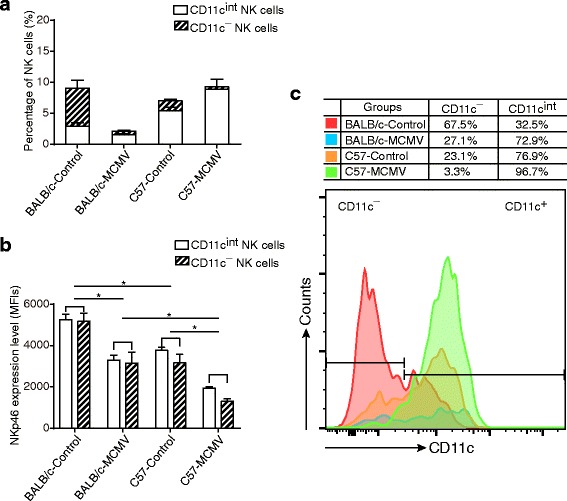
Distinct distribution of CD11c expression on NK cells of BALB/c and C57BL/6 mice. Control and MCMV groups of both BALB/c and C57BL/6 mice were generated as described above. Lungs samples were harvested and analyzed at 7 dpi. **a** The percentage of NK cells in control and MCMV groups is shown in the form of constituent ratio (*n* = 4 mice/group, results are representative of two independent experiments). Data are expressed as mean ± SD. **b** The expression levels of NKp46 in control and MCMV groups are shown. Data are expressed as mean ± SD (*n* = 4 mice/group, results are representative of two independent experiments). * *P* < 0.05. **c** Lung NK cells of each group are shown by histogram, based on their CD11c expression. The proportions of CD11c^−^ and CD11c^int^ NK cells in each group are shown in the table above the histogram

Comparison of the highly susceptible BALB/c mice with the resistant C57BL/6 mice indicated that, in the former control group, CD11c^−^ cells constituted the major proportion of NK cells (approx. 65% ~ 70%) in lung. After 7 dpi, however, the percentage of CD11c^−^ NK cells significantly dropped from 6.174% to 0.605%, while CD11c^int^ NK cells were only slightly decreased. Thus, CD11c^int^ cells become the dominant subpopulation of NK cells in MCMV lungs (Fig. 5a and c). In contrast, however, CD11c^int^ cells were the major NK cells (~75%) detected in the (uninfected) C57BL/6 controls; after the MCMV infection, nearly all the lung NK cells were CD11c^int^ (>95%; Fig. 5a and c).

The results above indicated the presence of a high proportion of CD11c^int^ cells in the lung NK cells after MCMV infection. CD11c has been reported as expressed on a subset of NK cells (known as B220^+^CD11c^int^); these cells were first termed ‘interferon-producing killer dendritic cells’ (IKDCs) and then, after refined characterization by subsequent studies, were defined as activated NK cells expressing B220 [17–19]. In order to explore whether these high proportion CD11c^int^ cells were B220^+^ NK cells, the NK cells of the lung and spleen were further analyzed and the process of change in B220^+^CD11c^int^ NK cells after MCMV infection was observed as well.

**Fig. 6:**
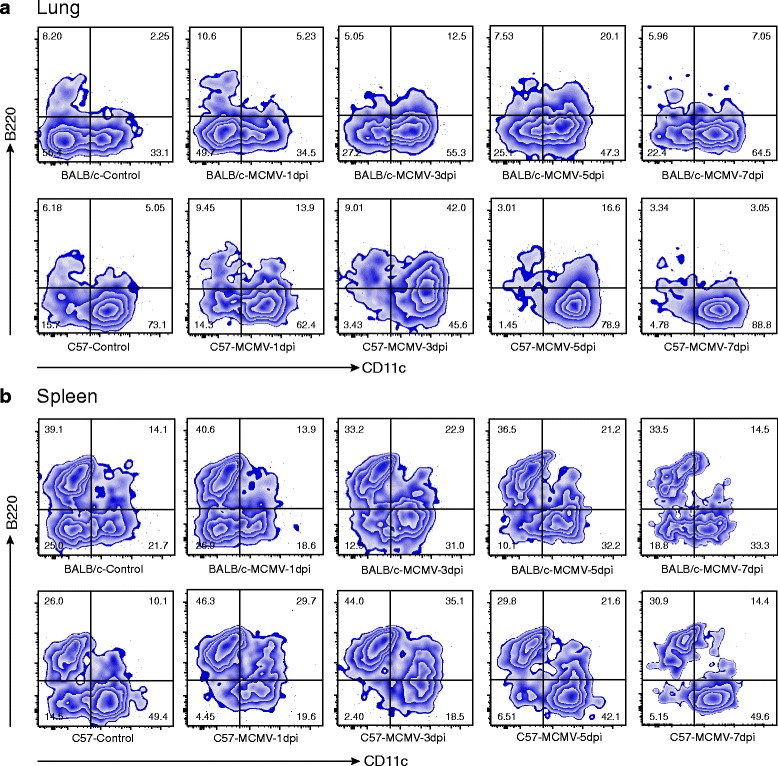
Distinct expression patterns of CD11c and B220 on NK cells of BALB/c and C57BL/6 mice. Control and MCMV groups of BALB/c and C57BL/6 mice were generated as described above. Lung and spleen samples were harvested and NK cells analyzed at 1, 3, 5 and 7 days post infection (dpi). **a** Lung NK cells from each group at different dpi are shown based on their expression of B220 and CD11c. B220^−^CD11c^−^ and B220^−^CD11c^int^ NK cells are the major NK cells in lungs of BALB/c and C57BL/6 mice, respectively. After MCMV infection, the proportions of B220^−^CD11c^int^ in BALB/c mice and B220^+^CD11c^int^ in C57BL/6 mice were significantly increased, with both peaking at 3 dpi. An obviously higher proportion of B220^+^CD11c^int^ was observed in C57BL/6 mice. **b** Spleen NK cells from each group at different dpi are shown based on their expression of B220 and CD11c. B220^+^CD11c^−^ is one of the major constituents of spleen NK cells, without obvious change after MCMV infection, the proportion of which was much higher than that in lung

The results for lungs (Fig. 6a) showed that, in the BALB/c mice, B220^−^CD11c^−^ cells constituted the major constituent of NK cells in the control group (~50%), followed by B220^−^CD11c^int^ cells. After the MCMV infection, B220^−^CD11c^int^ cells gradually emerged as the major constituent at 3 dpi. A slight upregulation of B220 was observed as well, contributing to a mild increase in the proportion of B220^+^CD11c^int^ cells at 3–5 dpi. However, the C57BL/6 mice showed obviously different expression patterns for B220 and CD11c. The B220^−^CD11c^int^ cells (60% ~ 70%) represented the major NK cells in the (uninfected) control group. After the MCMV infection, a significant upregulation of B220 was observed, with the proportion of B220^+^CD11c^int^ cells peaking at 3 dpi (~40%, much higher than that observed in the BALB/c mice). After that, the amount gradually dropped back to the control level. A similar trend was seen for the B220^−^CD11c^int^ and B220^+^CD11c^int^ NK cells in spleen (Fig. 6b). However, the main difference between the results from lung and spleen was related to the B220^+^CD11c^−^ cells, which accounted for less than 10% of the NK cells in lung, but represented an important component of the spleen (reaching up to 40% in that organ, wherein they showed no obvious change after the MCMV infection).

NKp46 (natural cytotoxicity receptor (NCR)1, CD335) is the first identified NCR and is conserved among all mammalian NK cells, regardless of their activation status and localization [20, 21]. In MCMV-infected mice (both BALB/c and C57BL/6), the expression level of NKp46 declined sharply, both in CD11c^−^ and CD11c^int^ NK cells, as compared to the control group. This decline in expression began at 3 dpi and was still ongoing at 7 dpi (Fig. 5b). The BALB/c mice showed higher NKp46 expression than the C57BL/6, for both the control group and MCMV group; however, no difference was observed between CD11c^−^ and CD11c^int^ NK cells when intragroup comparison was made.

## Discussion

An interesting finding of the present study is the generation of a large population of CD11c^int^ cells in the lung tissue in response to MCMV infection. Through screening with labeling antibodies, the CD11c^int^ cells were first identified as CD3ε^+^ T cells and then specified as CD8a^+^ CTLs. CD11c was first reported to be expressed by some CTLs in humans, suggesting their potential contribution to conjugate formation between CTL and target cells [22]. It was subsequently reported that CD11c^+^CD8^+^ T cells display signs of recent activation and are more efficient producers of IFN-γ, ultimately aiding in targeted cell lysis (as shown in vitro) and induction of viral clearance (as shown in vivo) [14].

Though the exact function of CD11c remains unknown, in different studies, CD11c^+^ CTL has been demonstrated to be able to exert both immunoregulation and effector functions [23]. In the present study, MCMV induced a large expansion of CD11c^int^CD8^+^ T cells (also known as CD11c^+^CD8^+^ T cells) at 7 dpi, which is consistent with the findings from the Beyer et al. [14] study of respiratory syncytial virus. However, in contrast to that previous study, the present study observed a higher proportion of lung CD11c^int^ CTLs (75 ~ 85% vs 40 ~ 50%). Meanwhile, no expansion of CD11c^−^ CTLs or of CD4^+^ T cells was observed, but MCMV-specific CTLs (mostly CD11c^int^ cells) were detected, indicating the possibility of CD11c^int^ CTLs playing a key role in anti-MCMV adaptive immune response. Interestingly, a recent study confirmed the proliferation of CD11c^+^CD8^+^ T cells induced by in vitro CMV antigen in human peripheral blood mononuclear cells [15]. The authors also reported that CD11c^+^CD8^+^ T cells represent an active effector phenotype and that the degree of CD11c expression in intra-tumor CD8^+^ T cells corresponds with their level of activation. Collectively, these studies indicate that CD11c expression is closely related with the function of CTLs involved in both viral clearance and tumor regression, and suggest its potential as a marker for the evaluation of host immune response and prognosis.

NK cells are essential for the control of a broad range of virus infections, including the widely studied CMV. The activation and function of NK cells depend on the balance of inhibitory and activating signals that are induced by the receptors expressed on their surfaces [24]. In the C57BL/6 mice, infection with m157-bearing MCMV led to rapid proliferation of Ly49H^+^ NK cells and better control of the viral infection. In contrast, the BALB/c mice, which lack Ly49H expression, are highly susceptible to MCMV infection [25]. Our result showed a decrease of NK cells in BALB/c at 7dpi, when C57BL/6-infected counterparts still displayed a high percentage of NK cells in lung, which was consistent with the failure of the former mouse strain to control MCMV. In addition, the distribution of CD11c expression was also different in NK cells of the two mouse lines (with and without Ly49H expression). B220^−^CD11c^−^ and B220^−^CD11c^int^ cells were found to be the major constituents of NK cells in the lungs in the control groups of the BALB/c and C57BL/6 mice, respectively. After the MCMV infection, the B220^−^CD11c^int^ cells proliferated and emerged as the major constituent in the BALB/c mice. Both BALB/c and C57BL/6 mice upregulated their B220 expression levels on B220^−^CD11c^int^ NK cells at 3–5 dpi, and a significantly higher proportion of induced B220^+^CD11c^int^ NK cells was observed in the C57BL/6 mice. These results indicate that B220^+^CD11c^int^ NK cells might represent a more effective type of NK cells, accounting for the observed resistance of C57BL/6 mice against MCMV infection.

Previous studies of the B220^+^CD11c^int^ NK cells have revealed that, as rapidly cycling cells, they can exert a highly effective cytotoxic activity and are highly effective secretors of IFN-γ upon stimulation [17, 19]. Yet, apart from Ly49H, it remains unknown whether or not the apparently discrepant expression patterns of CD11c and B220 (especially, the different performance of B220^+^CD11c^int^ NK cells) is also related with the anti-MCMV ability of NK cells in mice or has some other function.

As a commonly used DCs marker, CD11c has also been exploited as a target for in vivo depletion of DC populations [26, 27], namely by means of transgenic mice (i.e. Itgax-DTR-Tg mice expressing the diphtheria toxin (DT) receptor under the CD11c promoter) pretreated with DT before experimentation. In such studies, the results of depletion of CD11c^+^ subsets have been attributed largely to the DC-specific effect. However, results of our study showed that a portion of the NK cells and CD8^+^ CTLs can express CD11c. And the percentage of CD11c^int^ cells in both cell types can be greatly increased during MCMV infection. Thus, it is possible that targeting of CD11c-positive cells for depletion might result in depletions of CD8^+^ CTL or NK cell subsets as well. Further investigation is needed to determine the validity of such a hypothesis.

The difference of the lung anti-MCMV immune response that was found in the present study to exist between the two mice types examined involves not only the NK cells but also the CD8^+^ T cells response. A prominently higher level of CD11c^int^CD8^+^ T cells was detected in the C57BL/6 mice after MCMV infection, as compared to the BALB/c mice. It seems that C57BL/6 can induce, first, higher levels of NK cells and, then, higher levels of CD8^+^ T cells upon exposure to m157-bearing MCMV; these features may aid in bringing the infection under control as quickly as possible. In a previous study of Ly49H^+^ mice (compared to Ly49H^−^ mice) it was found that, after 6 dpi, the NK cells negatively regulated the anti-viral activity of CTLs, in order to suppress excessive immune response [1]. However, further study is still needed to confirm whether CD11c^int^ NK cells (the major NK cells represented), in particular, contribute to this process.

NKp46 is a type I transmembrane glycoprotein, which is involved in the control of various bacterial and viral infections [28, 29]. While NKp46 has been evidenced as an important mediator of the host response to influenza virus infection [30], little is known about its role in CMV infection and the findings in the literature are contradictory [28, 31, 32]. A recent in vitro study revealed that human NKp46 does not play a role in the anti-HCMV responses of decidual NK (dNK) cells [31]. Another study demonstrated a lack of difference in the early control of MCMV infection between NCR1^gfp/gfp^ mice and control mice [28]; yet, when a different group investigated *Noé* mice, which carry a point-mutation within the *NCR1* gene, they demonstrated a greater responsiveness of NK cells in vivo and a greater resistance phenotype to MCMV infection [32]. It seems that hyper-responsiveness of NK cells is associated with low NKp46 expression, as well as high *Helios* transcription [32, 33]. In the present study, lower expression of NKp46 on NK cells was found in C57BL/6 mice, compared to BALB/c mice with or without MCMV infection. After MCMV infection, decreased expression of NKp46 was detected in both mice lines. This is consistent with the results reported by Siewiera et al. [31], in which co-culture of dNK cells and HCMV-infected fibroblasts led to downregulation of NKp46. However, whether decline of NKp46 represents a manipulative strategy by CMV to evade the host immune system or a sign of increased reactivity remains unknown.

## Conclusions

The present study demonstrates that the vast majority of anti-MCMV CTLs induce CD11c expression which might witness their high activation status and potent effector functions for viral control.Intriguingly, BALB/c and C57BL/6 mice show distinct CD11c expression patterns on the surfaces of NK cells and CTLs, which might be linked with the distinctive anti-viral abilities of these immune cells. B220^+^CD11c^int^ NK cells might be a more effective type of NK cells participating in anti-MCMV infection. In vivo depletion strategies of DC populations by targeting the CD11c marker may result in depletions of CD8^+^ CTL or NK cell subsets as well. The association between the decline of cell surface NKp46 expression and anti-MCMV function is a question that should be investigated in future studies. The present study not only demonstrated CD11c expression change in MCMV-induced immune response, but has also provided future avenues of research into innate and adaptive immunity during the course of CMV infection.

## Additional file

Additional file 1 CD11c surface expression of CD4^+^ T cells. Lung CD4^+^ T cells in control and MCMV groups at 7 days post infection are shown by histogram, based on their CD11c expression. (PDF 122 kb)

## Abbreviations

cDCs: Conventional DCs
CMV: Cytomegalovirus
CTLs: Cytotoxic lymphocytes
DCs: Dendritic cells
dNK: Decidual NK
DT: Diphtheria toxin
LPS: Lipopolysaccharide
MCMV: Murine cytomegalovirus
MEF: Murine embryonic fibroblast
moDCs: Monocyte-derived DCs
NCR: Natural cytotoxicity receptor
NK cells: Natural killer cells
pDCs: Plasmacytoid DCs
PFU: Plaque forming unit
