# Supplementary figures and images for: Expression pattern of CD11c on lung immune cells after disseminated murine cytomegalovirus infection
Source: Virol J. 2017 Jul 18;14:132. doi: 10.1186/s12985-017-0801-x (PMC5516330; doi:10.1186/s12985-017-0801-x)

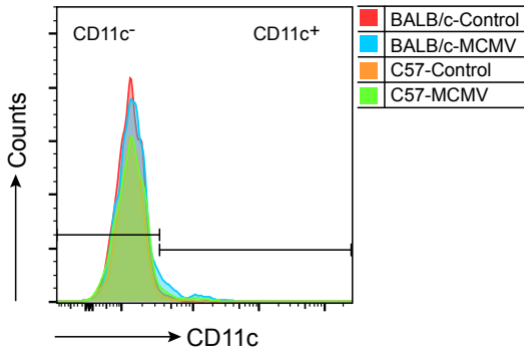

Supplement: Additional file 1 — CD11c surface expression of CD4+ T cells. Lung CD4+ T cells in control and MCMV groups at 7 days post infection are shown by histogram, based on their CD11c expression. (PDF 122 kb) [file 12985_2017_801_MOESM1_ESM.pdf]
